# Sentinel lymph node detection in early-stage breast cancer - Are technetium-99m-nanocolloid and superparamagnetic iron oxide diagnostically equivalent procedures? A propensity score matched real world data analysis

**DOI:** 10.1016/j.breast.2026.104766

**Published:** 2026-03-17

**Authors:** Ina Shehaj, Katharina Stuppy, Amelie Löwe, Paul Löwe, Christian Ruckes, Pia-Elisabeth Baqué, Mathias Schreckenberger, Yaman Degirmenci, Anne-Sophie Heimes, Antje Lebrecht, Andrea Helisch, Helmut Reber, Kathrin Stewen, Marcus Schmidt, Annette Hasenburg, Slavomir Krajnak

**Affiliations:** a Department of Obstetrics and Gynecology, University Medical Centre, Johannes Gutenberg University Mainz, 55131, Mainz, Germany; b Department of Gynaecology and Obstetrics, Johann Wolfgang Goethe University, Frankfurt am Main, Germany; c Department of Internal Medicine, Marienhaus Hospital, Mainz, Germany; d University Medical Centre of the Johannes Gutenberg University, Interdisciplinary Centre for Clinical Trials (IZKS), Mainz, Germany; e Department of Nuclear Medicine, University Medical Centre, Johannes Gutenberg University Mainz, Germany; f Department of Gynecology and Gynecological Oncology, Agaplesion Markus Krankenhaus, Frankfurt am Main, Germany

**Keywords:** Sentinel lymph node, 99mTc-nanokolloid, SPIO

## Abstract

Several studies have compared validated tracers, such as the radioactive tracer technetium-99m nanocolloid (99mTc-nanocolloid) and superparamagnetic iron oxide (SPIO), for sentinel node biopsy (SNB) in early-stage breast cancer (eBC). These studies mostly investigated the differences in detection rate and SNB-related adverse events. The aim of our study was to determine whether there are any crucial differences between the two procedures, including the duration of surgery and the number of detected sentinel lymph nodes (SN), which may affect patient treatment and the standard of care in eBC. All patients were treated at the University Medical Centre Mainz by certified breast surgeons. Two consecutive groups of patients were identified based on the injected tracer: Technetium Group (N = 517), patients treated from January 1, 2017 to December 31, 2019, with 99mTc-nanocolloid as tracer and SPIO Group (N = 456), patients treated from January 1, 2020 to December 31, 2022, with superparamagnetic iron oxide particles as ferromagnetic tracer. To avoid potential bias, we performed propensity score matching. After performing propensity score matching, the total surgery time was significantly shorter in the Technetium Group compared to the SPIO Group [64 (48 - 84) vs. 71.5 (58 - 98) minutes; p < 0.001]. Both groups did not differ regarding detection rates, or complications (p > 0.05). The median number of removed SN was 1.0 in the 99mTc-nanocolloid Group and 2.0 in the SPIO Group. In conclusion, our analysis showed that the use of 99mTc- nanocolloid for SNB resulted in shorter operation time and removal of less SN compared to the SPIO intervention.

## Highlights

• PSM analysis: 99mTC vs. SPIO for sentinel node biopsy in 973 breast cancer patients.
• Shorter surgery time in sentinel node biopsy with 99mTC compared to SPIO.
• Use of SPIO results in more nodes removed with no increase in complication rates.
• Sentinel lymph node detection rates were comparable for 99mTc and SPIO.

## 1 Introduction

Lymph node involvement remains one of the strongest predictors of long-term prognosis and survival [1,2] in breast cancer (BC) patients. The standard approach for axillary staging in patients with clinically node-negative early-stage breast cancer (eBC) is the sentinel node biopsy (SNB), although more recent data even show that omitting surgical axillary staging is not inferior to SNB in selected patients [[3], [4], [5]]. Since its establishment as the gold standard and the omission of conventional axillary dissection in the treatment of eBC, a significant decrease in the morbidity of node-negative patients with preservation of quality of life and without loss of diagnostic accuracy could be assured [6,7]. There are several techniques and tracers for SNB, each with its benefits and limitations. To date, the validated techniques in SNB worldwide include technetium-99m nanocolloid (99mTc-nanocolloid), and the dual technique, which combines 99mTc-nanocolloid and blue dye (Tc99/BD) and BD alone, followed later by superparamagnetic iron oxide (SPIO), indocyanine green (ICG) and contrast-enhanced ultrasound imaging (CEUS) [[8], [9], [10]].

With growing interest in optimizing SNB modalities, new approaches such as 99mTc- nanocolloid-tilmanocept (Lymphoseek), 99mTc combined with Rituximab, ICG-99mTc-nanocolloid, ICG mixed with BD (indigo carmine), ICG and Methylene Blue, and multimodal and hybrid techniques like preoperative CT lymphography (CTLG) with intraoperative SNB using fluorescence navigation and one-step nucleic acid amplification [[11], [12], [13], [14], [15], [16]] have been investigated. However, these new and modern tracers have not yet been validated, and further clinical studies are required to establish their application in the SNB.

Many studies have evaluated the differences among validated tracers, such as the radioactive tracer 99mTc-nanocolloid and SPIO, regarding detection rate and SNB-related adverse events. The non-inferiority of SPIO compared to 99mTc- nanocolloid ± BD was shown [17]. Liu et al. showed in a systemic review and meta-analysis of 19 studies, that no significant differences could be revealed in patient detection rates between the SPIO and standard method group (99m Tc- nanocolloid) [Hazard Ratio (HR) 1.00; 95% Confidence Interval (CI) 0.99–1.01; p = 0.21] [18]. Both SPIO and 99mTc-nanocolloid are approved for SNB in Germany, but in accordance with AGO recommendations, 99mTc-nanocolloid receives a higher recommendation of 2+, compared to SPIO's 1+ [19].

Many different essential aspects of the procedures were not thoroughly investigated.

Therefore, we performed this analysis of real-world data to examine whether there are any crucial differences between both procedures, such as: duration of surgery and number of detected and removed sentinel lymph nodes (SN), that could impact morbidity and treatment of patients, as well as the standard of care in eBC.

## 2 Materials and methods

### 2.1 Study population

In this retrospective observational analysis, we included patients with histopathologically verified BC, who underwent breast surgery including SNB at the Department of Gynecology and Obstetrics at the University Medical Centre Mainz. All our patients were clinically and ultrasonographically node-negative. Two groups of patients were identified based on the injected tracer: Technetium Group: patients treated from January 01, 2017 to December 31, 2019 with technetium-99m labelled nanocolloid (99mTc- nanocolloid) as a radioactive tracer, and SPIO Group: patients treated from January 01, 2020 to December 31, 2022 with SPIO as ferromagnetic tracer. Exclusion criteria included allergy to iron, dextran compounds or technetium, iron overload disease, pacemaker or ferrous metal-containing devices in the chest wall, pregnancy, and lactation.

Clinicopathological data such as age at initial diagnosis, body mass index (BMI), tumor size, nodal status, tumor localization, histologic grade of differentiation, estrogen receptor (ER), progesterone receptor (PR), human epidermal growth factor receptor 2 (HER2), Ki-67, treatment details, and type of injected tracer were obtained from the BC database of our department, medical and pathology reports. Clinicopathologic definitions of intrinsic breast cancer subtypes were used as follows [20].

1. Luminal A-like: ER-positive, PR high (>20%), Ki67 < 20%
2. Luminal B-like: ER-positive, PR low (<20%), Ki67 ≥ 20%
3. HER2-positive: HER2-positive (3+ on IHC or amplified on FISH (for 2+ IHC results)
4. Triple-negative: ER, PR and HER2-negative (HER2 1+, HER2+ and FISH not amplified)

### 2.2 Characteristics of the applied tracers

In the Technetium Group, essential data regarding the tracer were collected from the reports of the Department of Nuclear Medicine, University Medical Centre Mainz. 99mTc- nanocolloid was administered to the tumor area one day before the scheduled procedure, and lymphoscintigraphy was performed the day of injection. Intraoperatively, the SLN was identified using a hand-held gamma camera. The node with the highest pulse indication was designated as the SLN. Additionally, extra nodes were collected when their signal exceeded 10% of the pulse of the identified SN. The number of pulses in the identified SN were recorded each time in the operating protocol.

According to the technical information for Magtrace®, either 1 ml in 223 patients (May 2021 – December 2022) or 2 ml in 232 patients (January 2020 – April 2021) of diluted SPIO were injected into the interstitial tissue of the affected breast at least 20 min before the SNB. The institutional switch from 2 mL to 1 mL Magtrace® was guided by the available European approval and published clinical data indicating non-inferior SN detection compared with 2 mL [21,22].

Before the incision, the areola and breast, the injection site and the axillary area (hot spots) were measured using a handheld magnetometer. Polymer retractors and forceps were used to detect the SNs and avoid any interference with the magnetometer. All SPIO-SN were excised. Lymph nodes with less than 10% of the maximum SN count were defined as non-SN, and SNB was stopped when the residual activity in the axilla was less than 10%. In both methods, SNs and non-SNs were submitted separately for histopathological examination.

### 2.3 Outcomes

Our primary endpoint was to compare the surgery time between Technetium group and SPIO Group. *Total surgery time* was measured from the first skin incision to the final suture (skin-to-skin time). This definition was applied uniformly across the entire study cohort and remained unchanged throughout the study period. Surgery time was documented in the electronic medical record by the responsible surgical nurse and confirmed by the attending anesthesiologist as part of routine intraoperative documentation in our clinic.

Secondary endpoints were the success of both procedures, the number of SN detected by the surgeon intraoperatively, the number of removed SN as prescribed in the pathology report and postoperative complications. The success of both procedures was defined as positive intraoperative detection of SN and histological confirmation of the lymph node in the pathology report. Complications were defined as postoperative complications requiring a surgical intervention.

### 2.4 Statistical analysis

Due to the nonrandomized nature of the study design and the potential allocation biases arising from the retrospective comparison between both groups (99mTc- nanocolloid vs. SPIO) we performed a propensity score matching analysis.

**Fig. 1:**
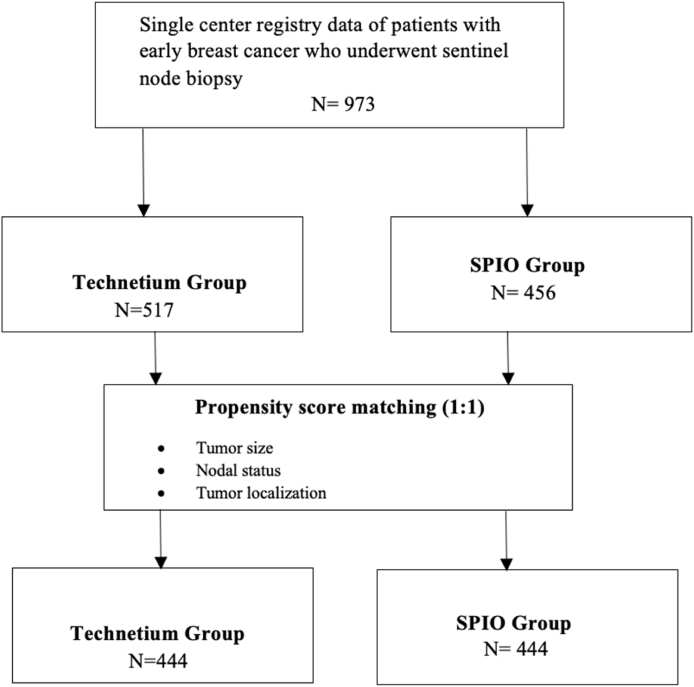
Consort flow chart of patient inclusion in the study.; SPIO, superparamagnetic iron oxide.

First, we identified the clinicopathological parameters, which significantly differed between both groups. Afterwards the concurrent effects of the statistically significant variables were eliminated. We determined the propensity score model (PSM) calculating propensity scores for each patient (Fig. 1) and conducted 1:1 matching using the greedy nearest neighbour matching method and a calliper width of 0.13 of the standard deviation of the logit of the estimated propensity score. Matching was performed by SAS Version 9.4, (SAS Institute Inc., Cary, NC, U.S.A.). The comparison between the two groups of patients was performed only in the propensity score-matched patients. After PSM, a multivariable linear regression model was used to assess the association between tracer type and surgery time, adjusting for type of breast surgery.

Statistical analyses were performed with SPSS statistical software program, version 27.0 V5 R (SPSS Inc, Chicago, IL, U.S.A.). A two-tailed p value < 0.05 was considered statistically significant. Patients’ characteristics were analyzed descriptively using median and interquartile range (IQR) for continuous data and absolute and relative frequencies for categorical data. Both groups were compared regarding clinicopathological characteristics and detection results of SN during SNB and after surgery, as described in the pathology report. The Mann-Whitney *U* Test and Pearson correlation were used to establish the statistical significance of the differences between both cohorts.

## 3 Results

### 3.1 Patient characteristics

Between 2017 and 2019 517 patients in the Technetium Group and between 2020 und 2022 456 patients in the SPIO Group were identified. Within the study cohort of 973 participants, patient and tumor characteristics were analyzed and outlined in Table 1.

**Table 1:** The baseline characteristics for the overall study cohort before Propensity Score Matching.

Characteristic	Technetium Group N = 517	SPIO Group N = 456	p value
Age (years), median(range)	60.0 (20 – 91)	60.0 (26 – 92)	0.699
Body mass index, median (range)	25.5 (16.6 – 55.3)	25.4 (14.2 – 54.5)	0.242

Tumor site, No (%)			0.506
Right breast	252 (48.7)	232 (50.9)	
Left breast	265 (51.3)	224 (49.1)	

Tumor localization, No. (%)			0.007
Upper outer quadrant	218 (42.2)	177 (38.9)	
Upper inner quadrant	95 (18.4)	73 (16.0)	
Lower inner quadrant	53 (10.3)	32 (7.0)	
Lower outer quadrant	80 (15.5)	70 (15.4)	
Central/retroareolar	31 (6.0)	37 (8.1)	
Multifocal/multicentric	40 (7.7)	66 (14.5)	

Pathological tumor size			0.002
Tis	21 (4.1)	22 (4.8)	
T1a	24 (4.6)	28 (6.1)	
T1b	84 (16.2)	75 (16.4)	
T1c	204 (39.5)	150 (32.9)	
T2	145 (28.0)	130 (28.5)	
T3	25 (4.8)	13 (2.8)	
T4	0 (0)	0 (0)	

Histology, No. (%)			0.109
DCIS	21 (5.6)	16 (3.5)	
IDC (NST)	376 (73.0)	363 (79.6)	
ILC	89 (17.3)	58 (12.7)	
Other∗	29 (5.6)	19 (4.2)	

Histological grading, No. (%)			0.681
Grade 1	85 (17.1)	66 (15.0)	
Grade 2	291 (58.6)	265 (60.1)	
Grade 3	121 (24.3)	109 (24.8)	

Intrinsic subtype, No. (%)			0.003
Luminal A-like	267 (53.7)	226 (51.7)	
Luminal B-like	154 (31.0)	118 (27.0)	
HER-2-positive	47 (9.5)	46 (10.5)	
Triple-negative	29 (5.8)	46 (10.5)	

Lymph node status			<0.001
N0	382 (73.9)	400 (87.7)	
N1	110 (21.3)	47 (10.3)	
N2	14 (2.7)	5 (1.1)	
N3	4 (0.8)	4 (0.9)	

Primary systemic therapy			<0.001
Yes	29 (5.6)	66 (14.5)	
No	488 (94.4)	389 (85.5)	

Abbreviations: DCIS, ductal cancer in situ; IDC (NST), invasive ductal cancer (nonspecific type); ILC, invasive lobular cancer; other∗ refers to mucinous breast cancer, medullary breast cancer, tubular breast cancer; SPIO, superparamagnetic iron oxide.

There were no significant differences between the cohorts in terms of age, BMI, tumor histology and histological grading (all p > 0.05). Significant differences were identified regarding nodal status (p < 0.001), tumor size (p = 0.002), intrinsic subtype (p = 0.003), tumor localization (p = 0.007) and primary systemic therapy (p < 0.001).

As shown in Table 2, both groups differed significantly regarding surgical procedures. Most patients in both groups (71.8% in the Technetium Group, 68% in the SPIO Group) were treated with simple wide local excision (WLE). The least frequently performed breast operation was oncoplastic breast-conserving surgery (OPBCS), 3.3% vs. 2.9%, respectively. In a few patients who initially underwent surgery for DCIS and were incidentally found to have BC, no further breast surgery was carried out. In those cases (Technetium Group 3,3% vs. SPIO Group 1,8%) only SNB was performed.

**Table 2:** Breast and axillary surgeries performed in both cohorts.

Characteristic	Technetium Group N = 517	SPIO Group N = 456	p value
Type of breast surgery			0.010
Simple wide local excision	371 (71.8)	310 (68.0)	
Oncoplastic breast-conserving surgery	17 (3.3)	13 (2.9)	
Modified Radical Mastectomy/	83 (16.1)	72 (15.8)	
Nipple-Sparing Mastectomy/Skin-sparing Mastectomy	29 (5.6)	53 (11.6)	
No Breast Surgery	17 (3.3)	8 (1.8)	

Type of axillary surgery			<0.001
SNB only	437 (84.5)	408 (89.5)	
Axillary Dissection in case of SNB failure	7 (1.3)	10 (2.2)	
Axillary Dissection in case of positive sentinel lymph nodes	68 (13.2)	21 (4.6)	
Targeted axillary dissection	0 (0)	11 (2.4)	
No axillary dissection performed, SNB failure	5 (1.0)	6 (1.3)	

Abbreviations: SPIO superparamagnetic iron oxide; SNB sentinel node biopsy.

SNB alone as axillary surgery was performed in 84.5 % of Technetium and in 89.5 % of SPIO patients. In a few cases, even though no sentinel could be detected, axillary surgery was terminated due to the multimorbidity and advanced age of the patients with low-risk BC (1.0% Technetium Group vs. 1.3% SPIO Group).

All patients were treated by four certified oncological breast surgeons according to the criteria of German Cancer Society.

Of the 973 assessed patients, 888 were included in the further analysis as part of a propensity score-matched sample (Fig. 1, Table 3).

**Table 3:** The baseline characteristics for the overall study cohort after propensity score matching.

Characteristic	Technetium Group N = 444	SPIO Group N = 444	p value
Age (years), median (range)	60.0 (20 – 91)	60.0 (26 – 92)	0.699
Body mass index, median (range)	25.5 (16.6 – 55.3)	25.4 (14.2 – 54.5)	0.242

Tumor site, No (%)			0.546
Right breast	219 (49.3)	228 (51.4)	
Left breast	225 (50.7)	216 (48.6)	

Tumor localization, No. (%)			0.159
Upper outer quadrant	191 (43.0)	175 (39.4)	
Upper inner quadrant	78 (17.6)	71 (16.0)	
Lower inner quadrant	38 (8.6)	32 (7.2)	
Lower outer quadrant	72 (16.2)	69 (15.5)	
Central/retroareolar	25 (5.6)	36 (8.1)	
Multifocal/multicentric	40 (9.0)	61 (13.7)	

Pathological tumor size			0.230
Tis	19 (4.3)	22 (4.8)	
T1a	24 (5.4)	27 (6.1)	
T1b	79 (17.8)	74 (16.7)	
T1c	186 (41.9)	149 (33.6)	
T2	106 (23.9)	130 (29.3)	
T3	17 (3.8)	12 (2.7)	
T4	0 (0)	0 (0)	

Histology, No. (%)			0.163
DCIS	19 (4.3)	16 (3.6)	
IDC (NST)	322 (72.9)	352 (79.3)	
ILC	76 (17.2)	57 (12.8)	
Other∗	25 (5.7)	19 (4.3)	

Histological grading, No. (%)			0.447
Grade 1	79 (18.5)	66 (15.3)	
Grade 2	247 (58.0)	262 (60.9)	
Grade 3	100 (23.5)	102 (23.7)	

Intrinsic subtype, No. (%)			0.072
Luminal A-like	236 (55.3)	226 (53.2)	
Luminal B-like	131 (30.7)	113 (26.6)	
HER-2-positive	34 (8.0)	42 (9.9)	

Triple-negative	26 (6.1)	44 (10.4)	

Lymph node status			0.371
N0	376 (84.7)	396 (87.8)	
N1	61 (13.7)	46 (10.4)	
N2	5 (1.1)	4 (0.9)	
N3	2 (0.5)	4 (0.9)	

Primary systemic therapy			<0.001
Yes	27 (6.1)	55 (12.4)	
No	417 (93.9)	388 (87.6)	

Abbreviations: DCIS, ductal cancer in situ; IDC(NST), invasive ductal cancer (nonspecific type); ILC invasive lobular cancer; other∗ refers to mucinous breast cancer, medullary breast cancer, tubular breast cancer, SPIO, superparamagnetic iron oxide.

The baseline characteristics of both cohorts after being matched are shown on Table 3. The application of neoadjuvant chemotherapy was the only baseline characteristic that differed significantly between both groups (Table 3).

The treatment characteristics regarding breast and axillary surgery in both cohorts after PSM are shown in Table 4. Both groups did not show any significant difference when compared regarding the surgical procedure. Interestingly, we noticed more cases of SNB failure in the SPIO Group compared to the Technetium Group (3.4 vs.1.1). However, this difference was not statistically significant.

**Table 4:** Breast and axillary surgeries performed in both cohorts after propensity score matching.

Characteristic	Technetium Group N = 444	SPIO Group N = 444	p value
Type of breast surgery			0.091
Simple wide local excision	328 (73.9)	304 (68.5)	
Oncoplastic breast-conserving surgery	10 (2.3)	13 (2.9)	
Modified Radical Mastectomy	67 (15.1)	69 (15.5)	
Nipple-Sparing Mastectomy/Skin-sparing Mastectomy	28 (6.3)	50 (11.3)	
No Breast Surgery	11 (2.5)	8 (1.8)	

Type of axillary surgery			0.840
SNB only	419 (94.4)	409 (92.1)	
Axillary Dissection in case of SNB failure	4 (0.9)	10 (2.3)	
Axillary Dissection in case of positive sentinel lymph nodes	20 (4.5)	20 (4.5)	
No axillary dissection performed, SNB failure	1 (0.2)	5 (1.1)	

Abbreviations: SPIO superparamagnetic iron oxide; SNB sentinel node biopsy.

### 3.2 Treatment outcomes regarding the applied tracer

As shown in Table 5a median (IQR) total surgery time was significantly shorter in the Technetium Group compared to SPIO Group after performing PSM [61.5 (47 – 81) vs. 71.0 (56 – 92) minutes; p < 0.001]. Multivariable linear regression analysis demonstrated, that the use of 99mTC as tracer still resulted in shorter operative time.

**Table 5a:** Comparison of the duration of surgery between the two cohorts after Propensity score matching.

Tracer/Type of surgery	Univariable analysis surgery time (minutes), median (IQR)	p value^a^	Multivariable analysis β coefficient (95% CI), p value^b^	p value^b^
Type of tracer		<0.001	10.767 (6.168 – 15.367)	<0.001
Technetium Group	61.5 (47.0 – 81.0)			
SPIO Group	71.0 (56.0 – 92.0)			

SPIO, superparamagnetic iron oxide; IQR, interquartile range; a p value Mann-Whitney *U* Test; b p value refers to the outcomes of ANOVA test; NA, not applicable.

We also examined the surgery time among both groups regarding different breast surgeries. The results are presented in Table 5b.

**Table 5b:** Duration of surgery regarding different breast surgeries among both groups after Propensity score matching.

Type of surgery	Technetium Group (median, IQR)	SPIO Group (median, IQR)	p value
WLE	57.5 (47 – 75)	67 (52-82)	0.020
OPBCS	85.5 (64 – 113)	98 (84 – 126)	0.613
MRM	71 (51 – 95)	80 (61.5 -106)	0.179
NSM/SSM	107 (81 – 147)	110 (78-139)	0.670

WLE, simple wide local excision; OPBCS, oncoplastic breast-conserving surgery; MRM, modified Radical Mastectomy; NSM, nipple-sparing Mastectomy; SSM, skin-sparing Mastectomy; SPIO, superparamagnetic iron oxide; IQR, interquartile range.

The success of the sentinel procedure, defined as the detection of SN intraoperatively and histological confirmation of lymph node(s), was 98.9% in the Technetium Group and 96.6% in the SPIO Group (Table 6). In the Technetium Group, the median number of SN detected and removed as assessed by the surgeon and confirmed by the pathologist was 1.0, regardless of the breast surgery. In the SPIO Group, the median number of SNs detected and removed by the surgeon was 2.0, except in the subgroup of patients who underwent SSM with SNB (1.0) (Table 6). SPIO doses (1 ml vs 2 ml) did not significantly affect neither the number of removed SN nor the duration of surgery (p values > 0.05; data not shown).

**Table 6:** Detection of sentinel and number of removed sentinel nodes regarding different breast surgeries among both matched groups.

		Technetium N = 444	Group			SPIO Group N = 444			p value
Detection of sentinel									
Yes (Nr. %)		439 (98.9)				429 (96.6)			0.017
Type of surgery	WLE + SNB	OPBCS + SNB	MRM + SNB	SSM + SNB	WLE + SNB	OPBCS + SNB	MRM + SNB	SSM + SNB	
Removed Sentinel lymphnodes surgeon (median)	1.0 (1-6)	1.0 (1-5)	1.0 (1-2)	1.0 (1-5)	2.0 (1-8)	2.0 (1-4)	2.0 (1-4)	1.0 (1-5)	<0.001
Removed Sentinel lymphnodes pathology (median)	1.0 (1-5)	1.0 (1-5)	2.0 (1-2)	1.0 (1-5)	2.0 (1-5)	2.0 (1-4)	2.0 (1-4)	2.0 (1-5)	<0.001

Abbreviations: WLE, simple wide local excision, OPBCS, oncoplastic breast-conserving surgery, MRM, modified Radical Mastectomy, SSM, skin-sparing Mastectomy; SNB, Sentinel lymph node biopsy; SPIO, superparamagnetic iron oxide.

The injection of both tracers was well-tolerated. No significant differences between the two groups regarding complications requiring surgery were noticed (Table 7).

**Table 7:** Comparison of complications of the procedure between matched groups.

	Technetium Group N = 444	SPIO Group N = 444	p value
None (%)	423 (95.3)	418 (94.1)	0.378
Postoperative hematoma	18 (4.1)	16 (3.6)	
Surgical-site infection	2 (0.5)	3 (0.7)	

## 4 Discussion

To our knowledge, this is the biggest real-world data analysis with 973 patients investigating the duration of surgery in both methods of SNB (99mTc- nanocolloid vs. SPIO). All patients were treated in the University Medical Centre Mainz by the same group of certified breast surgeons, which reduces bias in terms of surgical procedures and team experience.

Several studies compared 99mTc- nanocolloid and SPIO for SNB in breast cancer surgery [[23], [24], [25], [26]]. In the meta-analysis published by Pantiora et al., data synthesis of 20 comparative studies verified that SPIO performs comparably to 99mTc- nanocolloid ± BD, regardless of dose or injection site [27]. Besides comparing crucial parameters such as detection rates, concordance, and number of detected SNs, the duration of surgery was not part of the analysis. The largest trial included in this meta-analysis was the Nordic SentiMag trial with 338 patients with BC, who underwent 343 procedures (159 with 99mTc- nanocolloid vs. 184 with SPIO) [28]. However, this prospective cohort study was carried out in two different hospitals, one using 99mTc- nanocolloid and the other SPIO and the results reinforced the efficacy and safety of SPIO as an alternative to 99mTc- nanocolloid.

### 4.1 Surgical time

The primary outcome measure of our study was the comparison of the surgical time between both procedures (99mTc- nanocolloid vs. SPIO). In our study we showed that total surgical time was significantly 9.5 min shorter in the Technetium Group compared to the SPIO Group [61.5 (47 – 81) vs. 71.0 (56 – 92) minutes; p < 0.001]. Although the absolute reduction in operative time was approximately 9.5 min, this difference is clinically relevant. Operative duration has been identified as an independent risk factor for surgical site infection, with even incremental increases in surgical time associated with higher complication rates [29,30]. Extended surgical time is associated with higher rates of postoperative complications, as well as increased morbidity and even mortality and longer hospital stays [31,32]. From a health system perspective, a reduction of nearly 10 min per procedure may improve operating room efficiency, reduce cumulative anesthesia exposure, and increase daily surgical capacity, particularly in high-volume BC centers. Since some of the factors that increase surgical time are modifiable, using methods that may help to reduce surgical time and optimize work flow should be encouraged.

Therefore, we compared both methods of SNB. The analysis was performed in the propensity score matched patients, thereby reducing bias from covariates such as the type of breast surgery or the type of axillary surgery. The median surgical time did not differ between patients when comparing the first year of performing the procedure in both methods to the following two years (data not shown). Thus, the hypothesis that the experience of the surgeons using a new detection method for SNB can affect the duration of the surgery, could not be confirmed. When operative time was analyzed according to the type of breast procedure, the reduction in surgical duration associated with 99mTc was consistent across subgroups. The consistency of the effect across different surgical procedures suggests that the observed reduction in operative time is not driven by a single procedure type but reflects a general workflow advantage associated with 99mTC-guided SNB. Reduction of surgical time in the treatment of eBC was also investigated in other trials in terms of cost-effectiveness and treatment process optimization [27,33,34].

A pilot study by Shams et al. investigated the effects of 99mTc- nanocolloid and SPIO on care process optimization, reimbursement, surgical time, and patient comfort in BC patients undergoing SNB. The supraparamagnetic iron lymphatic tracer (Magtrace) localization shortened the preoperative care pathway [ (5.4 ± 1.3 min) vs. (82 ± 20 min); *p* < 0.0001]. This study showed, that the overall surgery time was not significantly longer when SPIO was used for SNB (Magtrace: 74 min vs. Tc99: 71 min; p = 0.891) [33]. Interestingly, Shams et al. reported that Magtrace had a cost-saving effect compared to the use of Tc99 [33].

While previous studies, such as the pilot study by Shams et al., did not demonstrate a significant difference in overall operative time between SPIO and 99mTC, differences in study design, sample size, and workflow organization may explain these discrepancies. Our findings, derived from a PSM cohort, suggest that in routine clinical practice 99mTC may confer a meaningful reduction in surgical time.

### 4.2 Detection rates and removed sentinel nodes

In our study, the success rates were 98.9% for 99mTc and 96.6 % for SPIO, showing a similar effectiveness in terms of SN identification. Previous studies investigated the detection rates of both methods [23]. Our observation in the 99mTc- nanocolloid Group corresponded well to the results of large prospective studies where the SN identification rate was around 97% [35].

At the same time, in a large meta-analysis by Mok et al., SPIO showed a 97.4% identification rate, ranking second-best (after 99mTc-nanocolloid) in terms of detection rates [8]. In agreement with our analysis, all studies demonstrated non-inferiority of the SPIO technique compared to 99mTc [10,23,24,26].

Interestingly, the increased fatty tissue in the breast in elderly patients may cause a decreased lymphatic flow. It is also suggested that the replacement of lymph nodes by fatty tissue decreases the capacity of lymph nodes to retain the radioactive colloid [36]. Indeed, Cox et al. observed in their study a significant association between BMI and detection rate (p = 0.042). They reported that each one-year increase of age or one-unit of BMI reduced the chances of success by approximately 5% [36]. However, both cohorts of our study did not differ significantly regarding age and BMI.

Other studies suggest that 99mTc-based methods may detect a slightly smaller number of SNs compared to SPIO. Thill et al. reported in the SentiMag study with150 patients included, that an average of 1.8 SLNs (range: 1-9 nodes) was detected per patient using 99mTc- nanocolloid and 1.9 SLNs (range: 1-9) with SPIO, suggesting a slight increase of removed nodes with SPIO [26]. In addition, the Nordic SentiMag trial found that SPIO identified more SN per patient compared to the standard technique (p < 0.001) [25]. Our results confirm previously mentioned studies, showing that with 99mTc-nanocolloid a lower number of SN is removed compared to SPIO (median SN: 1 vs. 2, Table 4). The higher number of removed SN in the SPIO Group possibly lead to a higher morbidity.

### 4.3 Advantages and disadvantages of both procedures

SPIO is safe, simple to store, and easy to apply. The main side effect reported is dermo-pigmentation, which typically decreases over time [37]. Indeed, in the Nordic SentiMag Trial, depigmentation occurred in 35.5% of patients and remained slightly paler and smaller after one year in 21% of patients and in 8.6% of patients after 15 months [25]. In concordance with other clinical trials, we did not observe any allergic reactions in our study cohort [25,38].

However, a significant limitation of SPIO is that it should be avoided in patients where magnetic resonance imaging (MRI) will later be used to evaluate treatment response. It is also not suitable for patients undergoing breast MRI surveillance. The use of SPIO in these cases can interfere with MRI interpretation, limiting its diagnostic effectiveness. Van Haaren et al. demonstrated that even 1 ml SPIO tracer used for SN procedure impaired the evaluation of breast MRI at the tracer injection site beyond one year of follow-up [39]. In patients for whom breast MRI plays a central role in treatment monitoring or long-term surveillance, including those receiving neoadjuvant chemotherapy or young high-risk mutation carriers, potential MRI interference should be considered when selecting SPIO as a tracer.

On the other hand, 99mTc- nanocolloid also has some disadvantages. Its limited availability and high costs make access challenging for many hospitals [10]. The procedure necessitates a nuclear medicine department. However, the injection of 99mTc-nanocolloid can also be performed in nuclear clinics outside the hospital, where the surgery will be performed. The use of radioactive materials in the operating room used to generate significant concern about radiation exposure However, radiation exposure to operating room personnel, pathologists, and operative equipment during a SNB using 99mTc-nanocolloid is minimal [40]. Several studies have concluded that moderate activities of technetium-99m labelled tracer are administered to the patient, and the radiation risk to the patient is consequently low relative to that from many other medical exposures [41].

In our study, we demonstrated a comparable risk of surgical complications between both methods.

One limitation of our study was the retrospective and monocentric design. Another limitation was the lack of data on quality of life and long-term survival. However, a notable strength of our study is including a large number of consecutive, clinically node-negative breast cancer patients treated by the same group of board-certified breast surgeons. We also used propensity matching to minimize potential bias.

In conclusion, our study provides valuable insights into the comparison of surgical time between 99mTc- nanocolloid and SPIO in SNB, showing that SPIO prolongs the surgical time without increasing the complication rate. Furthermore, the study confirmed the safety and feasibility of both procedures for the SNB.

## CRediT authorship contribution statement

**Ina Shehaj:** Writing – original draft, Visualization, Validation, Resources, Methodology, Formal analysis, Data curation, Conceptualization. **Katharina Stuppy:** Data curation. **Amelie Löwe:** Writing – review & editing, Data curation. **Paul Löwe:** Data curation. **Christian Ruckes:** Writing – review & editing, Software, Methodology, Formal analysis. **Pia-Elisabeth Baqué:** Writing – review & editing, Data curation. **Mathias Schreckenberger:** Writing – review & editing. **Yaman Degirmenci:** Writing – review & editing. **Anne-Sophie Heimes:** Writing – review & editing. **Antje Lebrecht:** Writing – review & editing. **Andrea Helisch:** Writing – review & editing. **Helmut Reber:** Writing – review & editing. **Kathrin Stewen:** Writing – review & editing. **Marcus Schmidt:** Writing – review & editing. **Annette Hasenburg:** Writing – review & editing. **Slavomir Krajnak:** Writing – review & editing, Validation, Supervision, Resources, Project administration, Methodology, Investigation, Conceptualization.

## Informed consent statement

Not applicable.

## Institutional Review Board statement

Written informed consent was obtained from all patients, and all clinical investigations were conducted ethically in accordance with ethical and legal standards and in consideration of the Declarations of Helsinki. The study was conducted in accordance with the Declaration of Helsinki and approved by the Institutional Review Board of Ethics Committee of Rhineland-Palatinate, Germany (no. 837.139.05).

## Data availability statement

The main data supporting the findings of this study are available within the paper and its Supplementary Information. The datasets used and analyzed during the current study are available from the corresponding author on reasonable request.

## Funding

This research did not receive any specific grant from funding agencies in the public, commercial or not-for-profit sectors.

## Declaration of competing interest

The authors declare the following financial interests/personal relationships which may be considered as potential competing interests: A.-S. Heimes received honoraria from Pfizer Pharma GmbH, Roche Pharma AG, Daiichy Sankyo GmbH, Medupdate GmbH, and Streamedup! GmbH. K. Almstedt received speaker honoraria from Roche Pharma AG, Pfizer Pharma GmbH, and AstraZeneca. K. Stewen received honoraria from Astra Zeneca, Roche Pharma AG, StreamedUp! GmbH, and Pierre Fabre. I. Shehaj received funding and travel reimbursement from Novartis Pharma GmbH, AstraZeneca, Olympus and AURIKAMED. S. Krajnak received speaker honoraria from Roche Pharma AG and Novartis Pharma GmbH Germany, research funding from Novartis Pharma GmbH Germany and travel reimbursement from PharmaMar and Novartis Pharma GmbH Germany. A. Hasenburg received honoraria from AstraZeneca, Celgen, GSK, LEO Pharma, MedConcept GmbH, Med update GmbH, Medicultus, Pfizer, Promedicis GmbH, Softconsult, Roche Pharma AG, Streamedup! GmbH, and Tesaro Bio Germany GmbH. She is a member of the advisory board of AstraZeneca, GSK, LEO Pharma, PharmaMar, Promedicis GmbH, Roche Pharma AG, Tesaro Bio Germany GmbH, MSD Sharp and Dohme GmbH. M. Schmidt reports personal fees from AstraZeneca, BioNTech, Daiichi Sankyo, Eisai, Gilead, Lilly, MSD, Novartis, Pantarhei Bioscience, Pfizer, Roche, and SeaGen outside the submitted work. Institutional research funding from AstraZeneca, BioNTech, Eisai, Genentech, German Breast Group, Novartis, Palleos, Pantarhei Bioscience, Pierre Fabre, and SeaGen. In addition, Marcus Schmidt has a patent for EP 2390370 B1 issued and a patent for EP 2951317 B1 issued. All other authors declare that they have no conflicts of interest. If there are other authors, they declare that they have no known competing financial interests or personal relationships that could have appeared to influence the work reported in this paper.
